# A Content Analysis of Alcohol Marketing on Instagram: Examining Its Contribution to an Australian Aquatic Alcogenic Environment

**DOI:** 10.3390/ijerph23020217

**Published:** 2026-02-09

**Authors:** Gemma Crawford, Renee N. Carey, Nicola D’Orazio, Jonine Jancey, Justine E. Leavy

**Affiliations:** 1 Collaboration for Evidence, Research and Impact in Public Health, Curtin University, Perth, WA 6102, Australia; g.crawford@curtin.edu.au (G.C.); renee.carey@curtin.edu.au (R.N.C.); nicola.dorazio@curtin.edu.au (N.D.); j.jancey@curtin.edu.au (J.J.); 2 Faculty of Health Sciences, Curtin School of Population Health, Curtin University, Perth, WA 6102, Australia

**Keywords:** alcohol, social media, young people, injury, drowning, advertising

## Abstract

**Highlights:**

**Public health relevance—How does this work relate to a public health issue?**

- The commercial determinants of health, specifically the alcohol industry, influence health and wellbeing.
- Alcohol use in and around waterways contributes to fatal and non-fatal drowning in young people.

**Public health significance—Why is this work of significance to public health?**

- Social media alcohol advertising normalises alcohol use in aquatic locations.
- Social media platform alcohol advertising policies are commonly circumvented.

**Public health implications—What are the key implications or messages for practitioners, policy makers and/or researchers in public health?**

- Future public health action should focus on stricter advertising restrictions on social media to mitigate the impact of alcohol marketing, particularly for young people.

**Abstract:**

The alcohol industry uses social media platforms to normalise alcohol consumption in aquatic locations, which can lead to injury and drowning. This research investigated alcohol content posted on Instagram to determine marketing strategies and the extent to which aquatic locations and activities were featured. The Australian Instagram accounts of five alcohol brands were identified across beer, spirits, and cider. The 20 most recent posts were captured for analysis. Each image and caption were saved and coded to examine (1) ‘What marketing characteristics are used by alcohol brands on Instagram?’; and (2) ‘Which aquatic locations and activities featured in the posted Instagram content?’. Ninety-nine posts comprising 176 individual images were identified and analysed. Alcoholic beverages were shown in 53.4% of images. Aquatic locations were observed in 21.6% and aquatic activities in 17.0%. The use of aquatic locations and activities varied significantly by brand (*p* < 0.001). Advertising themes included associating alcohol with hobbies (29.6%) and mateship (22.7%). Only 21.6% of images contained an age restriction or warning; all accounts employed some form of age-gating. Social media platforms provide alcohol industry actors the opportunity to increase their exposure to young people. Brands used imagery that associated alcohol with fun and mateship and promoted the use of alcohol in aquatic locations.

## 1. Introduction

Alcohol consumption in and around waterways contributes to fatal and non-fatal drowning in Australia. For example, 2024 data [1] indicated that alcohol was involved in fatal drownings across: lakes and dams (17%); ocean, harbour, rock and cliff locations (16%); creeks and rivers (15%); swimming pools (14%); and beaches (4%). Aquatic environments present particular concerns for alcohol-related harm due to the specific risks associated with water. Alcohol impairs cognitive function, judgment, and motor coordination, while also affecting thermoregulation and the diving reflex—physiological factors that significantly increase drowning risk [2,3].

However, activities in and around water vary in their inherent risk profiles [4,5]. Swimming and water entry present immediate risks of immersion incidents. Research on boating-related drowning specifically identifies alcohol as a contributing factor in a substantial proportion of incidents [6,7], while broader evidence links recreational swimming deaths to alcohol consumption [2]. In the most recent Australian secondary school students’ use of alcohol and other substances survey, around one in five young people (21%) who were current drinkers reported that their last alcoholic drink was consumed in a location that included recreation areas such as the beach [8].

While individual-level factors such as intoxication are well-documented contributors to drowning risk, less attention has been paid to the commercial practices that influence alcohol consumption in aquatic settings. Further, while research has established that alcogenic environments facilitate alcohol consumption [9], the specific contribution of alcohol marketing, including on social media, to normalising alcohol consumption in aquatic settings has not been systematically examined [4,10]. Understanding how commercial practices construct normative expectations about drinking in these environments is essential for effective public health responses. Accordingly, this study examines how alcohol marketing on Instagram contributes to what we have termed *an aquatic alcogenic environment* [11,12].

### 1.1. Alcohol Marketing and the Commercial Determinants of Health

Public health research increasingly shows how commercial determinants of health (CDoH) influence health and wellbeing [13,14], recognising that an individual’s health and behaviours exist within a broader system that includes physical and social environments, political and economic policies, and commercial actors [13]. It has been argued that the system works to enable, rather than constrain, commercially driven health harms [14] and illustrates that rather than an individual’s (in)ability to make “healthy choices” or take “personal responsibility”, health is shaped by mechanisms of corporate power [13].

Alcohol industry actors exert substantial political and social influence in Australia [15]. Industry actors frame alcohol harms as an issue of excessive consumption by a small minority, contrasted with the “responsible” practices of the majority of drinkers [16]. This framing is strengthened using preference-shaping and constituency-building or coalition-management tactics [17]. To illustrate, alcohol companies undertake corporate social responsibility activities, such as funding organisations like ‘DrinkWise’ [18], which purportedly seeks to foster a “healthy drinking culture” in Australia [19], despite no conclusive evidence to suggest that they reduce harmful drinking [20].

The alcohol industry spends over $100 million annually on advertising in Australia [21] as well as broader marketing activities such as investing in sponsorship of sports, events, and individuals [22]. Alcohol marketing normalises consumption, increases brand loyalty and reinforces positive perceptions of alcohol [15]. Despite industry claims to the contrary [23], evidence suggests that exposure to alcohol advertising and promotion, particularly among young people, can lead to intentions to consume alcohol and an increased likelihood of harmful drinking [15,21,24]. Marketing receptivity research demonstrates that positive affect toward alcohol marketing, including brand identification and ownership of branded merchandise, predicts drinking behaviour more strongly than mere exposure [25,26].

### 1.2. Alcogenic Environments and Place-Based Risk

The concept of alcogenic environments provides a framework for understanding how environmental factors shape alcohol consumption and related harms. These environments have been defined as settings characterised by high alcohol consumption, easy access to alcohol, and permissive alcohol marketing and supply, which collectively normalise drinking behaviours [9]. Attributes, including alcohol affordability, aggressive marketing, and outlet density, have been linked to hazardous alcohol consumption [27,28,29]. Alcogenic environments have been identified as a distal risk factor for heavy and harmful alcohol use, particularly among young people [30].

Historical place-based literature has suggested that alcohol consumption in different settings exposes drinkers to different risks, and that these risks become greater with continued alcohol use [31,32]. Such approaches move beyond individual-level explanations to acknowledge that health is shaped by the interactions between individuals and their physical, social, and symbolic environments [33]. For example, more recent literature suggests that alcohol consumption is related to environmental characteristics, including accessibility, social context, entertainment provision, advertisement placement, and premise décor, with young drinkers describing how “everything is telling you to drink” in certain environments [9].

In this context, the concept of “social worlds” provides a useful framework for understanding how marketing constructs drinking cultures around specific leisure activities. Social worlds have been described as loosely bounded groups characterised by shared commitments, practices, and norms that are generated and reinforced through interaction among members [34]. Importantly, social worlds of alcohol consumption are framed by the settings where people drink, the products consumed, and the technologies used in doing so. This conceptualisation shifts analytical focus from individual drinkers to the interpersonal influences that sustain drinking, a perspective relevant to understanding how social media marketing constructs socially expected contexts for alcohol consumption.

### 1.3. The Aquatic Alcogenic Environment

Building on this literature, we have proposed the concept of an *aquatic alcogenic environment* to describe settings where alcohol marketing, environmental properties, and social practices converge to normalise consumption in and around waterways [4,11,12]. This suggests that, for many young people, particularly in jurisdictions such as Western Australia, with significant access to waterways and an outdoor recreation culture, social activities occur in and around beaches, rivers, and pools. Coastal and beach locations often feature licensed venues, drinking-focused events, and high rates of alcohol-related harm among young people [12].

The concept extends place-based risk frameworks by identifying three mechanisms through which aquatic settings may amplify alcohol-related harm. Water-related leisure carries symbolic meanings associated with relaxation, freedom, and escape from everyday constraints [35], affective associations that alcohol marketing frequently exploits [36]. In addition, activities in and around aquatic environments involve embodied risks that are significantly heightened by alcohol consumption [3,37], with recent research finding that more than half of young people surveyed indicated they had participated in ‘drinking and swimming’ in the last year [38]. Moreover, peer influence operates strongly in recreational aquatic environments, with research suggesting that alcohol-related risk in these settings is substantially shaped by social networks [4,11,39]. The aquatic alcogenic environment thus represents a specific instantiation of broader place-based risk, where environmental cues, social norms, and marketing messages create spaces where alcohol consumption is sanctioned and made routine, but in settings with inherent physical dangers.

### 1.4. Alcohol Marketing and Social Media

Social media has transformed alcohol marketing, enabling brands to reach wider audiences, encourage user interaction, and blur boundaries between commercial and organic content [40,41]. Social media amplifies the reach of alcohol marketing [42], with a recent review of social media alcohol advertising exposure in Australia reporting that on average, 765 alcohol advertisements were shared weekly on Meta platforms, primarily by alcohol brands [43]. In addition to these more traditional advertisements, alcohol promotion on platforms such as Meta’s Instagram occurs through branded pages, influencer marketing and user-generated content, often solicited by alcohol companies through the promotion of hashtags and competitions [21].

Social media’s ubiquity and increasing sophistication of digital alcohol marketing make regulation more challenging, in part due to the variety of techniques through which alcohol brands can target (predominantly young) consumers, meaning that advertising is increasingly difficult to regulate [21]. In Australia, alcohol advertising is self-regulated through the Alcohol Beverages Advertising Code (ABAC) [44]. However, evidence suggests that the Code is regularly contravened and fails to capture problematic alcohol advertisements [21,45], suggesting limited effectiveness [46]. Additionally, while many social media platforms have policies to regulate the promotion of harmful products such as alcohol, research shows that these are commonly circumvented [47,48]. The literature suggests that governments have been slow to direct social media companies to enforce their policies systematically and have provided limited oversight [49].

### 1.5. Symbolic Consumption and Social Contexts

Understanding how alcohol marketing operates on social media platforms requires attention to the symbolic dimensions of consumption and the social contexts within which drinking practices are embedded. Digital marketing employs affective strategies to build brand equity [50] and encourage consumption [51]. These themes resonate particularly with young people, who are engaged in identity formation and are preoccupied with personal image [52].

Unhealthy commodity marketing research has demonstrated that symbolic consumption strategies are central to the promotion of unhealthy products such as alcohol, tobacco, and gambling, where brands are situated alongside valued lifestyle activities to transfer positive cultural meanings to products [41,53,54,55,56]. Research on young people’s engagement with alcohol marketing on social media has further illuminated these meaning-making processes. Critchlow and colleagues found that young people viewed alcohol marketing on social media as portraying consumption positively, considered it an everyday part of online experiences, and recognised that branding holds symbolic value that facilitates identity construction and peer interaction [25]. When alcohol brands associate their products with aspirational lifestyles and aquatic leisure, they harness associations that may create social acceptance of such consumption in inherently risky settings. In previous research with young people, consuming alcohol before or while participating in aquatic activities was more common among those who more frequently recalled seeing alcohol advertising and engaging with alcohol brands and products on social media [38].

### 1.6. Summary

Taken together, this literature suggests that social media platforms have emerged as significant sites for the construction and performance of alcohol-related social worlds. The present study extends this literature by examining how Australian alcohol brands construct a specific social world organised around aquatic leisure, a context of public health significance given the established relationship between alcohol consumption and drowning risk.

The research is part of a larger study building evidence on alcohol industry advertising in aquatic locations, recognising that alcohol use and participation in activities in and around waterways contribute to fatal and non-fatal drowning. Specifically, the research sought to answer the following questions: (1) ‘What marketing characteristics are used by alcohol companies on Instagram?’ and (2) ‘Which (if any) aquatic locations and activities feature in the Instagram content posted by alcohol companies?’.

## 2. Materials and Methods

### 2.1. Brand and Post Selection

Five alcohol brands were identified across drink categories (beer, spirits, cider). Brand selection was informed by: systematic outdoor advertising research identifying brands having advertised in aquatic locations in metropolitan Perth, Western Australia [12]; evidence from prior research that the brand’s social media advertising targeted young Australians aged 16 to 25 [57]; brands commonly consumed in Western Australia; and, popular drink categories for young people nationally (beer and spirits) [8]. Brands represent diverse market segments within the Australian alcohol industry, including local/craft beer, global beer, international cider, and multinational spirits. The identified Australian Instagram accounts were:

(i) Gage Roads Brewing Company (@gageroadsbrewco), a Perth-based craft brewery with a strong association with Western Australian beach culture;
(ii) Corona Australia (@coronaextra_au), a Mexican beer brand distributed in Australia by Asahi and recognised for beach-lifestyle marketing;
(iii) Pirate Life Brewing (@piratelifebeer), an Adelaide-founded craft beer now owned by Carlton & United Breweries, oriented to younger consumers with sponsorship of music and cultural events;
(iv) Somersby Australia (@somersbyaustralia), a cider brand owned by Carlsberg, marketed toward both men and women with lifestyle-focused imagery; and
(v) Smirnoff Australia (@smirnoffaustralia), a vodka and premixed spirits brand owned by Diageo. In Australia, Smirnoff is marketed through music festival sponsorships.

A study-specific Instagram account was created and used to access the public accounts of these five brands. Each account was accessed at the commencement of the Australian Spring season (1 September 2024). Account details and the 20 most recent posts from each account were captured and saved to a relevant folder. The decision to analyse the 20 most recent posts per account was made to account for variability in posting frequency across included brands and to ensure data sufficiency for analysis. It yielded an average number of posts and data consistent with or exceeding comparable studies of alcohol brand social media content [58,59,60]. For posts that included multiple static images (i.e., carousel images), each image was captured.

Video posts (i.e., ‘Instagram Reels’, n = 626) and those appearing on the accounts but posted by another Instagram account were excluded (n = 1). Exclusion was based on several considerations: (1) the established coding framework was developed for static image analysis and would require adaptation for video content; (2) video content presents distinct analytical challenges including duration variability and the difficulty of capturing representative ‘moments’ for coding; and (3) focusing on static posts allowed for systematic comparison with earlier studies on alcohol advertising imagery [58,61].

The following account details were captured at the date of access: account name, total number of posts and reels, number of followers and accounts following, age-gating, and links posted in the account bio. Information for each post captured: date of access, date posted, hyperlink, number of likes and comments, and caption.

### 2.2. Coding Process

A coding framework captured: (a) aquatic locations and features depicted in each image; (b) use of advertising themes (e.g., humour, sports, partying), (c) visual cues (e.g., tags, use of emojis), (d) advertising devices (e.g., use of animation) and (e) marketing activities (e.g., sponsorship, partnerships). This framework was informed by relevant advertising codes and the extant literature. Coding categories for advertising themes were adapted from an analysis by Pettigrew and colleagues of alcohol advertising across multiple media, which identified humour, mateship, and lifestyle associations as prevalent strategies [61]. Categories relating to marketing activities, including sponsorship, influencer partnerships, and branded merchandise, drew on emerging literature on digital marketing strategies [25,40]. Aquatic-specific categories (locations and activities) were developed from our research mapping alcohol advertising around waterways, with young people about alcohol and drowning risk, and alcohol-related drowning risk factors [4,5,11,12,38,62]. Categories for visual cues (emojis, tags) followed prior research [63,64], recognising these as distinctive traits of platform-native content.

Table 1 provides an overview of the coding categories.

The coding framework was initially tested by two coders (BR and GW) who analysed several posts together in a training session with members of the study team (RNC and JEL). Disagreements were resolved by discussion between the coders and the study team, and refinements were made. The coders then analysed a random sample of 10 posts separately, with any disagreements resolved by discussion. The average level of agreement for these posts was 90.0%. Following refinement, the remaining posts were analysed by one of two coders (BR and GW) using a spreadsheet developed in Microsoft Excel. Any further uncertainties were discussed with the study team and resolved by agreement.

### 2.3. Statistical Analysis

Descriptive statistics summarised post characteristics overall and by brand. Pearson Chi-square tests were used to assess differences in key image characteristics across brands, with *p* < 0.05 as the level of statistical significance. Stata version 18 was used for all statistical analyses.

## 3. Results

The accounts had followers ranging from 9483 to 46,385 and published between 241 and 3528 posts (Table 2). A total of 99 posts were captured for analysis. Posts had varied reach, with an average of 66.7 likes (Standard Deviation (SD) = 73.2, median = 43, range 4 to 406) and 1.7 comments (SD = 4.9, median = 1, range 0 to 45) per post. These posts included 31 static-image carousels, totalling 176 images available for analysis.

### 3.1. Images

Table 3 presents the characteristics of images overall and by alcohol brand. Alcohol products (the alcoholic beverage) and brands were shown to varying degrees, with Gage Roads posts most likely to include branded merchandise (e.g., clothing and hats) and posts from Corona most likely to feature branded alcoholic beverages (Table 2). Alcoholic beverages were shown in over half of images (n = 94, 53.4%), most commonly beer (n = 51, 29.0%), spirits or premixed drinks (n = 24, 13.6%) and cider (n = 9, 5.1%), and non-alcoholic or ultra-low alcohol beverages were featured in 8 images (4.6%).

Aquatic locations were observed in 21.6% of all images (n = 38), most frequently beaches (n = 25, 14.2%) and oceans (n = 12, 6.8%) (Table 2). Significant brand differences were observed in the use of aquatic locations, with more than two-thirds of Corona images (n = 14, 70.0%) featuring a beach, whereas 14.9% of Gage Roads (n = 7) and 15.0% of Corona (n = 3) images featured the ocean (*p* < 0.001). Aquatic activities were featured/presented in 17.0% of all images (n = 30), with surfing featured in 8.5% of images (n = 15) and relaxing around waterways in 6.8% of images (n = 12). Again, there were significant brand differences, with no images posted on the Pirate Life, Somersby, or Smirnoff accounts depicting aquatic activities (*p* < 0.001). Surfing was a common Gage Roads image (n = 12, 25.5%) and relaxing around waterways was frequently seen in Corona images (n = 6, 30.0%).

Over two-thirds of images (n = 122, 69.3%) depicted a person or group, with posts from Corona and Smirnoff particularly likely to feature a group (Table 3). Approximately one-quarter of all images (n = 41, 23.3%) featured people who appeared to be aged under 25 years, and over half of all Smirnoff images (n = 22, 53.7%) featured young people (*p* < 0.001). The apparent gender of people varied, with Gage Roads (n = 18, 38.3%) and Pirate Life (n = 17, 37.8%) more likely to feature males, and Somersby (n = 7, 30.4%) and Smirnoff (n = 12, 29.3%) more likely to contain females.

The use of advertising themes such as humour, mateship, and lifestyle varied (Table 2). Brand differences were seen for the use of mateship (*p* = 0.036), which was most common in Corona images (n = 9, 45.0%); happiness or fun in association with alcohol (*p* = 0.006), which was most common in images posted by Smirnoff (n = 17, 41.5%); and the association of alcohol use with hobbies and interests (e.g., yoga, skiing or music) (*p* = 0.003) was most common in images posted by Somersby (n = 12, 52.2%) and Smirnoff (n = 19, 46.3%). The use of advertising devices also varied, with logos and branded colours a particular feature of images posted by Gage Roads (n = 42, 89.4%), Somersby (n = 19, 82.6%), and Smirnoff (n = 35, 85.4%) but used less frequently by Pirate Life (n = 23, 51.1%) (*p* = 0.001). The use of sponsorships or partnerships (e.g., partnerships with music festivals such as Splendour in the Grass) was a particular characteristic of images posted by Smirnoff (n = 20, 48.8%), whereas it was used relatively infrequently or not at all by the other brands (*p* < 0.001).

### 3.2. Captions

Alcoholic beverages were mentioned in under half of all captions (n = 42, 42.4%) (Table 4). Beer was the most frequently mentioned beverage (n = 27, 27.3%), with four captions (4.0%) mentioning non-alcoholic or ultra-low-alcohol beverages.

Most captions did not mention an aquatic location (n = 89, 89.9%) or aquatic activity (n = 92, 92.9%). Surfing was mentioned in one in five captions (n = 4, 20.0%) accompanying Corona posts (*p* = 0.049), and the beach was mentioned in 10.5% of Gage Roads captions (n = 2) and 15.0% of Corona captions (n = 3) (*p* = 0.377). One-third of captions (n = 33, 33.3%) mentioned alcohol in relation to hobbies and/or interests such as entertainment and beauty, and this was particularly common in captions posted by Smirnoff (n = 13, 65.0%) and Pirate Life (n = 9, 45.0%) (*p* = 0.001). Smirnoff captions linked celebrities and sportspeople to their brand (n = 5, 25.0%) (*p* = 0.037), and captions posted by both Pirate Life (n = 9, 45.0%) and Smirnoff (n = 13, 65.0%) were significantly more likely to reference the sponsorship of events (e.g., Pirate Life sponsorship of Port to Port football event) or collaboration between brands (*p* < 0.001).

Emojis were used in just over half of all captions (n = 51, 51.5%). The use of emojis was particularly common in captions posted by Gage Roads (n = 16, 84.2%) and Pirate Life (n = 16, 80.0%), and uncommon in captions posted by Corona (n = 1, 5.0%) (*p* < 0.001). Brand differences were also observed in the use of tags in captions (*p* < 0.001). Overall, one-quarter (n = 25, 26%) of captions tagged another user, of which seven (7.1%) were individuals, and 18 (18.2%) were companies or brands. Pirate Life was most likely to tag other companies or brands in their captions (n = 10, 50.0%). Tags were also commonly used by Gage Roads, who tagged both individuals (n = 3, 15.8%) and companies or brands (n = 5, 26.3%) in their captions.

All accounts employed age-gating, and all but one (Gage Roads) displayed age restrictions on their homepage. However, age restrictions were used inconsistently. Only 38 (21.6%) images contained an age restriction or warning; the majority of Corona (n = 19, 95.0%) and Somersby (n = 19, 82.6%) images included a stated age restriction. No images from the other three accounts contained an age warning (*p* < 0.001). No captions from any post contained an age warning or restriction.

## 4. Discussion

Social media is a key marketing strategy of industries selling harmful products, such as alcohol [40]. This study investigated content posted on the Instagram accounts of five popular Australian alcohol brands, exploring the extent to which aquatic locations and activities were featured, as well as the advertising themes and marketing strategies used in the 20 most recent posts from each account. Collectively, the five brands had over 135,000 followers and posted over 8000 images. The content contained elements that appeared to produce meaning and enhance appeal, construct an aquatic alcogenic environment, extend traditional marketing reach, engage young people and utilise gendered marketing patterns. Finally, there was some evidence of “water-washing” [10] (activities such as community sponsorship in and around waterways or content related to zero or low alcohol products that link aquatic environments to these products), and likely non-adherence to the ABAC.

Analysis of content across the five brands revealed two overarching narratives underpinning alcohol promotion in aquatic contexts. *Lifestyle integration* frames alcohol as an integral component of aquatic leisure, operating through imagery that naturalises the co-presence of alcohol and water (e.g., Corona’s beach-relaxation scenes, Gage Roads’ surf-culture associations, or Smirnoff’s festival-adjacent aquatic settings). *Social connection* links alcohol to associations of mateship, fun, and shared experience, positioning alcohol as essential to social currency and identity formation (e.g., Corona emphasised relaxed group bonding, Pirate Life highlighted the craft beer community, and Smirnoff portrays energetic social scenes). These themes construct aquatic environments as spaces in which consumption is not only acceptable but also expected, aligning with broader marketing strategies [41,65]. These themes are explored further below.

### 4.1. Producing Meaning and Enhancing Appeal

Alcohol brands appeared to invest in content aligned with leisure, social, and cultural practices; hobbies; and locations associated with aquatic environments, particularly the beach and the ocean. Consistent with existing scholarship [36], brands located their products within socially desirable habitus, often associating alcohol with mateship (e.g., Corona), happiness and fun (e.g., Smirnoff) or hobbies and interests (e.g., Somersby). However, the study found differences between brands in how they portrayed alcohol consumption and aquatic locations. The beach and ocean settings frequently depicted in Corona imagery, for example, draw on cultural scripts of escape and freedom, themes that alcohol advertising frequently promises to deliver [36]. Similarly, the association of surfing with beer brands like Gage Roads draws on cultural meanings around adventure, authenticity, and masculinity [66]. These findings illustrate how brands are not merely depicting consumption but transferring the positive cultural meanings of aquatic leisure to their products.

Such orientation demonstrates how branded goods function as symbolic resources through which consumers construct and signal valued identities [67,68] and how marketing can acculturate alcohol consumption within specific social contexts [25,51]. The concept of ‘sociability’, association for its own sake, independent of instrumental purposes [69], offers additional insight into how this marketing operates. This framework has been applied to contemporary drinking contexts, demonstrating how consumption enhanced feelings of belonging in leisure settings, reinforcing social bonds through shared ritual practices [70]. When alcohol brands embed their products in aquatic leisure contexts, they tap into pre-existing associations between drinking and sociability, entrenching products within established cultural scripts of social connection and collective enjoyment.

### 4.2. Constructing the Aquatic Alcogenic Environment

More than one in five images analysed featured aquatic locations. This association merits consideration, given established links between alcohol consumption in such settings and elevated drowning risk [3], particularly for the activities depicted (e.g., boating, swimming, surfing). Our findings reinforce how marketing contributes to the cultural legitimation of alcohol consumption in these environments [71], through several mechanisms, exploiting the symbolic and affective associations of water-related leisure evident across brands.

By saturating social media feeds with alcohol-waterway associations (e.g., drinking on beaches and boats, or while fishing), brands make the relationship between alcohol and waterways ubiquitous. Brands also create positive framing by consistently associating alcohol consumption in and around waterways with desirable states (happiness, relaxation), thereby constructing this behaviour as desirable [38]. Finally, by linking alcohol to social identities and lifestyle markers valued by young people (e.g., spending time with friends), brands frame drinking in aquatic contexts as a natural expression of self, contributing to what has been termed “intoxigenic social identities”, which frame “alcohol consumption … as a normative practice among youth” [72], reinforcing drinking in aquatic contexts as a shared, socially sanctioned practice [73]. When alcohol consumption around waterways is framed as a positive, identity-congruent behaviour, young people may be less likely to recognise or respond to the genuine dangers such consumption introduces [3]. The aquatic alcogenic environment thus operates not merely as a physical risk context but as a lens through which consumption is culturally legitimated and socially rewarded.

### 4.3. Extending Traditional Reach

A proportion of images included groups of people, pointing to the socially influenced nature of drinking practices, warranting attention, as research suggests alcohol risk in and around waterways is influenced by peers [4]. Social media platforms have emerged as significant sites for the construction and performance of alcohol-related social worlds [34,72,74,75]. Our findings suggested that alcohol brands actively construct social worlds organised around aquatic leisure, characterised by shared visual codes (sunset beaches, surfboards, coastal landscapes), normative expectations (that alcohol accompanies water-based leisure), and identity resources (the “beach lifestyle” consumer).

Placement of socially oriented content demonstrates the effective peer networking strategies employed on social media, including virality, engagement and the use of influencers [76,77]. As an example, there was evidence of co-branding strategies by tagging other companies or influencers [78]. Such strategies enable brands like Pirate Life and Gage Roads to expand their presence across social media. These approaches also blur the line between organic (non-paid) and commercial content, increasing the appeal of alcohol to demographics who may be more influenced by the perception of authentic branding [76]. Content analysis of alcohol-related posts on Facebook and Instagram found that imagery featuring groups received significantly more engagement [79,80] and this pattern was evident in our findings, where socially oriented content appeared designed to maximise engagement and sharing, thereby extending brand reach through peer networks.

### 4.4. Engaging Young People

Nearly one-quarter of the images analysed contained individuals who appeared to be under 25, with Smirnoff using this strategy most frequently. This finding raises questions, given evidence that young people are both more susceptible to advertising influence [24] and at increased drowning risk [5], with more than one in nine drowning deaths in 2023/2024 involving those aged 15–24 years [1]. While companies argue that they do not promote their products to children, their presence on social media demonstrates that these platforms are viable channels for reaching young audiences and promoting early brand adoption, thereby circumventing rules and guidelines restricting product advertising to children [43]. This is reinforced by the finding that many posts utilised casual visual storytelling techniques (e.g., tags and emojis), which may hold greater appeal for younger audiences [64].

One action often called for is better age-gating or age verification. All accounts in this study used age-gating to prevent those under 18 from viewing their posts. However, research suggests that age-gating and verification alone are ineffective controls to prevent young people from alcohol marketing exposure [81], in part because online content exists in a significantly cluttered offline marketing landscape, requiring additional controls [77].

### 4.5. Utilising Gendered Marketing Patterns

Content contained characteristics that appeared to enhance appeal to specific identities, a tactic reported in the broader literature [65]. Brands displayed differentiated approaches to depicting men and women, reflecting findings consistent with prior research suggesting that alcohol marketing on social media employs gendered strategies [58,82].

Around one in four images in our study featured males. Gage Roads and Pirate Life were more likely to feature males, often in active contexts such as surfing, aligning with research identifying the association between alcohol marketing and hegemonic masculinity [65,66]. Humour and mateship themes were commonly used by these brands. Prior research suggests these themes appeal particularly to young males, a demographic known for risk-taking [83]. In contrast, brands like Somersby and Smirnoff were more likely to feature women. Around one in three posts in our study used captions aligned with marketing strategies documented in previous research as appealing to women, including references to lifestyle content for women, such as beauty and fashion, a strategy reported in other research on Instagram content [58].

These differentiated approaches reflect the gendered marketing logics documented across the broader literature, whereby brands strategically align products with existing cultural constructions of masculinity and femininity [65,82,84]. From a drowning prevention perspective, these gendered patterns merit scrutiny. By embedding alcohol in masculine-coded aquatic activities, brands may contribute to drinking norms that heighten risk for young men, who account for the majority of alcohol-related drowning deaths [1,35,66,85].

### 4.6. Applying “Water-Washing” Strategies

Marketing, in the form of merchandise and sponsorship, was evident in around half of the posts by Smirnoff. Such marketing represents industry tactics to link products to “good corporate citizen” activities [20]. As an example, we found evidence of what we have termed “water-washing” [10], corporate social responsibility strategies, including sponsorship (e.g., of music festivals); a tactic documented in other research [61]. Findings from the broader literature [86] suggest that these activities are often harder for young people to distinguish as alcohol marketing.

A small number of posts promoted zero or no-alcohol products (ZAP), most often posted during Dry July, aligning with wellness and “sober curious” trends [87]. However, marketing these products alongside traditional alcohol brands raises public health concerns. Often, ZAPs are situated as “healthier” alternatives, which may downplay the risks associated with alcohol consumption and create a misleading sense of safety, problematic in contexts of aquatic environments. These have also been argued as an industry “stealth” marketing tactic, providing a potential gateway for alcohol brands to maintain their relevancy [88].

### 4.7. Perceived Inconsistencies with Regulatory Guidance

Despite guidelines indicating that alcohol advertising will not contain activities linked to risk-taking, our findings identified content that may be inconsistent with the intent of the ABAC [44] (though we note our analysis does not constitute a formal regulatory compliance assessment). By way of illustration, some posts appeared inconsistent with Standard 3B (responsibility towards minors, e.g., depicting those who appear under 25 years of age), with one in four posts featuring individuals who appeared to be under 25. We identified posts featuring alcohol in relaxation contexts, warranting investigation in relation to Standard 3C (responsible depiction of the effects of alcohol, e.g., alcohol aids relaxation). Further, several posts contained content that appeared relevant to Standard 3D (alcohol and safety, e.g., consumption related to the control of a boat or while swimming). Gage Roads and Corona frequently paired alcohol imagery with beach and ocean contexts, and around 10% contained specific activities, such as surfing. These findings are problematic given that one in three drowning deaths in Australia in 2023/2024 were linked to recreating and swimming [1], including boating [6,7]. Despite known safety risks, content that may tacitly endorse drinking near water suggests a regulatory gap [89].

The regulatory challenges identified in this Australian context reflect broader international concerns. The World Health Organization has highlighted significant gaps in alcohol marketing regulation across borders, with a recent report noting that 2018 data suggested almost half of countries had no regulation for internet (48%) or social media (47%) alcohol marketing [90]. The WHO SAFER initiative identifies advertising restrictions as a ‘best-buy’ for reducing alcohol harm [6,91], yet the sophistication and cross-border nature of digital marketing pose significant challenges for national regulatory frameworks [92]. The WHO European Region has documented how alcohol marketing adapts to digital platforms faster than regulations can respond [90], with only 14 of 53 Member States reporting bans on internet- and social-media alcohol marketing [93]. Our findings contribute to an international evidence base documenting the specific strategies that alcohol brands deploy on digital platforms [94], strategies that are consistent across jurisdictions but that existing regulatory frameworks appear ill-equipped to address.

### 4.8. Recommendations

Future research could explore the impact of alcohol advertising in aquatic environments across a broader range of social media platforms and extend analysis to video-based content (e.g., Instagram Reels). These platforms are increasingly important components of alcohol marketing strategies, particularly for reaching young audiences. TikTok presents additional considerations in relation to its younger user demographic, algorithm-driven content discovery, and short-form video format [95]. Recent research examining user-generated alcohol content on TikTok and Instagram found high rates of pro-brand sentiment and evidence of undisclosed marketing [59]. A similar analysis focused on aquatic themes would contribute valuable insights into how video formats are employed to promote alcohol in aquatic environments.

Consideration of a broader range of brands, including additional multinational brands and brands that do not specifically target aquatic contexts, would enable comparison of marketing strategies across environments. Such expanded sampling would strengthen an understanding of whether the patterns identified in this study are transferable across Australian and global alcohol contexts. Comparing alcohol advertising between aquatic and non-aquatic environments may also be instructive. Investigating the long-term effects of exposure to such advertising on young people’s behaviours in and around waterways would be beneficial. There is also a need to better understand how gender and age influence young people’s social worlds and their engagement with online alcohol marketing featuring aquatic environments.

Future public health action could focus on stricter advertising restrictions and bans on social media to mitigate the impact of alcohol marketing, particularly for young people [4,77,89,96]. Drawing on international evidence and the SAFER framework [90,91], this may include statutory restrictions extending broadcast advertising standards to social media platforms. Content regulations could prohibit the depiction of alcohol consumption in conjunction with water activities, vehicles, or other risky behaviours, thereby extending existing regulatory provisions to explicitly include aquatic environments. Greater platform accountability measures could compel social media companies to implement robust age verification, with independent monitoring and sanctions for non-compliance, and to restrict influencer marketing by requiring clear disclosure of commercial relationships and prohibiting alcohol promotion by influencers whose audiences include a substantial proportion of users under 18 years. Cross-border regulatory frameworks consistent with mechanisms established under the WHO Framework Convention on Tobacco Control [49] should be investigated.

In the Australian context, this includes addressing documented limitations in the ABAC scheme, which has been identified as ineffective broadly [97], and particularly inadequate for controlling digital alcohol advertising [43,77,98]. Monitoring the potential positive impacts of Australia’s recent social media bans for those under 16 years provides an opportunity to understand public health implications for alcohol consumption. Advocacy efforts by public health and drowning prevention organisations could draw more heavily on existing and future Code standards related to health and safety (including Standards 3C and 3D) to highlight contravening content, lodge more formal complaints about it, and encourage greater government oversight. Finally, the findings point to an emerging opportunity to examine how the industry employs “water-washing” tactics, including the sponsorship of sports and other activities in aquatic environments.

### 4.9. Strengths and Limitations

To our knowledge, this is the first study to examine social media in the context of alcohol and aquatic environments, which is important for guiding recommendations of the Australian Water Safety Strategy 2030 [37]. Several limitations are noted. Posts represent only a snapshot of marketing activities by a small number of brands viewed by a segment of the population. Collectively, alcohol brands had substantial reach in the Australian market; although they do not constitute a representative sample of all alcohol brands, focusing on brands relevant to the WA setting provides local context. Advertising on Instagram is only one way that companies promote alcohol products; assessing content across other social media platforms may have yielded additional insights. However, Instagram is a particularly popular platform used by young people [99].

The exclusion of video-based content represents an important limitation. We acknowledge that short-form video content has become increasingly dominant on social media platforms [100] and that alcohol brands are actively utilising this format. Video content employs strategies including dynamic visual storytelling, audio branding, and extended narrative engagement that static posts cannot capture. Future research should examine alcohol marketing in Reels and other short-form video formats (e.g., TikTok), which are potentially more impactful marketing channels for reaching young audiences [59]. Demographic information on individual followers of the company accounts was unavailable, so we were unable to assess the characteristics of users who interact with brand content [78]. Furthermore, platform algorithms may have influenced which content was visible to researchers; this effect was minimised by using a new account to view content. Finally, we note that the sample represents a temporal snapshot and that the volume and type of alcohol advertising may vary, resulting in an under- or over-estimate of portrayals of aquatic locations and activities [96], which may not capture seasonal variations.

## 5. Conclusions

Alcohol companies in Australia employ sophisticated strategies to promote their products, often going beyond traditional advertising. This study contributes to the literature on commercial determinants of health, with findings that reinforce the role of corporate practices and marketing strategies in shaping public health outcomes. Empirically, it provides the first systematic examination of social media alcohol marketing in aquatic contexts, documenting the prevalence, characteristics, and brand-level variations in such content. Theoretically, it advances the concept of the aquatic alcogenic environment as a specific instantiation of place-based alcohol risk, extending the alcogenic environment framework by identifying how marketing contributes to normalisation in settings with inherent physical dangers and highlighting the mechanisms through which place-based traits interact with commercial practices to shape health outcomes. Policy implications from this study relate to the broad failure of self-regulation to effectively curb harmful advertising, highlighting the need for stringent policies and enforcement regarding alcohol marketing, particularly on social media, to decrease young people’s risks for alcohol-related harm, including in and around aquatic environments. Social media alcohol marketing continues to target young people and promotes activities known to pose risks for drowning, requiring greater attention across injury, alcohol and commercial determinants of health spheres of scholarship and public health action.

## Figures and Tables

**Table 1:** Example coding framework categories.

Coding Type	Category	Sample Codes	
**Accounts**	Account details	Brand/Account name Following/Followers Posts/Posts (last year)	Reels/Reels (last year) Account age restriction Age-gating
**Posts**	Post details	Post ID Date posted/Date taken Hyperlink	Username Likes Comments
	Content of post	Alcohol beverage Alcohol brand	Pictures/words/both Transcription of post
**Captions**	Content of caption	Alcohol beverage	Content of caption
	Visual cues	Emojis	Tags
**Posts and captions**	Aquatic features	Aquatic location	Aquatic activity
	Advertising themes	Alcohol and safety Depiction of young people Alcohol and success Partying Mateship/friendship	Humour Happiness Lifestyle activities (e.g., socialising, holidays) Hobbies and interests (e.g., workshops, food, fishing) Gendered roles (e.g., makeup)
	Advertising devices	Branding elements (e.g., logos)	Characters/mascots
	Marketing activities	Competitions, prizes, giveaways Vouchers, offers, rebates	Celebrities/sportspeople Sponsorships and partnerships (e.g., music festivals)

**Table 2:** Account metrics for selected alcohol brand accounts on Instagram, overall and by brand.

Account Metrics	Overall (n = 176)	Gage Roads (n = 47)	Corona (n = 20)	Pirate Life (n = 45)	Somersby (n = 23)	Smirnoff (n = 41)	Mean (SD) ^a^
Number of followers	137,704	39,682	31,592	46,385	9483	10,562	27,540.8 (16,832.2)
Number of accounts followed	5529	1008	668	3127	565	161	1105.8 (1169.6)
Total number of posts	8882	3528	2161	2488	464	241	1776.4 (1396.6)

^a^ SD = Standard Deviation.

**Table 3:** Characteristics of selected images posted by alcohol brands on Instagram, overall and by brand.

Image Characteristics (n, %)	Overall (n = 176)	Gage Roads (n = 47)	Corona (n = 20)	Pirate Life (n = 45)	Somersby (n = 23)	Smirnoff (n = 41)	*p* Value ^a^
**Alcohol product or brand shown**							<0.001
On merchandise	43 (24.4)	20 (42.6)	3 (15.0)	12 (26.7)	0 (0.0)	8 (19.5)	
As sponsorship or branding	17 (9.7)	0 (0.0)	0 (0.0)	1 (2.2)	1 (4.4)	15 (36.6)	
On alcohol packaging	67 (38.1)	17 (36.2)	15 (75.0)	11 (24.4)	8 (34.8)	16 (39.0)	
Logo only	20 (11.4)	5 (10.6)	0 (0.0)	2 (4.4)	10 (43.5)	3 (7.3)	
**Aquatic location featured**							<0.001
Beach	25 (14.2)	10 (21.3)	14 (70.0)	1 (2.2)	0 (0.0)	0 (0.0)	
Ocean	12 (6.8)	7 (14.9)	3 (15.0)	2 (4.4)	0 (0.0)	0 (0.0)	
Pool	1 (0.6)	1 (2.1)	0 (0.0)	0 (0.0)	0 (0.0)	0 (0.0)	
**Aquatic activity featured**							<0.001
Surfing	15 (8.5)	12 (25.5)	3 (15.0)	0 (0.0)	0 (0.0)	0 (0.0)	
Boating or manual watercraft	2 (1.1)	1 (2.1)	1 (5.0)	0 (0.0)	0 (0.0)	0 (0.0)	
Diving or snorkelling	1 (0.6)	0 (0.0)	1 (5.0)	0 (0.0)	0 (0.0)	0 (0.0)	
Relaxing around waterways	12 (6.8)	6 (12.8)	6 (30.0)	0 (0.0)	0 (0.0)	0 (0.0)	
**Features a person or people**	122 (69.3)	30 (63.8)	18 (90.0)	25 (55.6)	15 (65.2)	34 (82.9)	0.066
One person	52 (29.6)	18 (38.3)	3 (15.0)	13 (28.9)	7 (30.4)	11 (26.8)	0.008
Group of people	70 (39.8)	12 (25.5)	15 (75.0)	12 (26.7)	8 (34.8)	23 (56.1)	
Young people (aged < 25 yrs)	41 (23.3)	8 (17.0)	4 (20.0)	3 (6.7)	4 (17.4)	22 (53.7)	<0.001
Male(s)	45 (25.6)	18 (38.3)	2 (10.0)	17 (37.8)	0 (0.0)	8 (19.5)	<0.001
Female(s)	29 (16.5)	4 (8.5)	3 (15.0)	3 (6.7)	7 (30.4)	12 (29.3)	
Group of mixed gender	37 (21.0)	4 (8.5)	12 (60.0)	2 (4.4)	5 (21.7)	14 (34.2)	
**Advertising themes**							
Mateship	40 (22.7)	8 (17.0)	9 (45.0)	9 (20.0)	2 (8.7)	12 (29.3)	0.036
Humour	5 (2.8)	3 (6.4)	0 (0.0)	1 (2.2)	1 (4.4)	0 (0.0)	0.383
Happiness or fun	39 (22.2)	5 (10.6)	6 (30.0)	7 (15.6)	4 (17.4)	17 (41.5)	0.006
Association with lifestyle	32 (18.2)	6 (12.8)	7 (35.0)	5 (11.1)	5 (21.7)	9 (21.9)	0.145
Association with hobbies and interests	52 (29.6)	9 (19.2)	6 (30.0)	6 (13.3)	12 (52.2)	19 (46.3)	0.003
**Advertising devices**							
Logos, branded colours, or other branding	134 (76.1)	42 (89.4)	15 (75.0)	23 (51.1)	19 (82.6)	35 (85.4)	<0.001
Characters or mascots	4 (2.3)	0 (0.0)	0 (0.0)	4 (8.9)	0 (0.0)	0 (0.0)	0.018
**Marketing activities**							
Celebrities or sportspeople	9 (5.1)	1 (2.1)	0 (0.0)	2 (4.4)	0 (0.0)	6 (14.6)	0.028
Sponsorship or collaboration	33 (18.9)	4 (8.7)	0 (0.0)	7 (15.6)	2 (8.7)	20 (48.8)	<0.001

^a^ Differences between brands assessed using Chi-Square test.

**Table 4:** Characteristics of captions associated with posts by alcohol brands on Instagram, overall and by brand.

Caption Characteristics (n, %)	Overall (n = 99)	Gage Roads (n = 19)	Corona (n = 20)	Pirate Life (n = 20)	Somersby (n = 20)	Smirnoff (n = 20)	*p* Value ^a^
**Alcoholic beverage mentioned**							<0.001
Beer	27 (27.3)	6 (31.6)	9 (45.0)	11 (55.0)	0 (0.0)	1 (5.0)	
Spirit/premix	7 (7.1)	0 (0.0)	0 (0.0)	0 (0.0)	0 (0.0)	7 (35.0)	
Cider	4 (4.0)	0 (0.0)	0 (0.0)	0 (0.0)	4 (20.0)	0 (0.0)	
Non-alcoholic	4 (4.0)	3 (15.8)	0 (0.0)	1 (5.0)	0 (0.0)	0 (0.0)	
**Aquatic location featured**							0.377
Beach	6 (6.1)	2 (10.5)	3 (15.0)	1 (5.0)	0 (0.0)	0 (0.0)	
Ocean	1 (1.0)	0 (0.0)	0 (0.0)	1 (5.0)	0 (0.0)	0 (0.0)	
Other (undetermined)	2 (2.0)	0 (0.0)	0 (0.0)	1 (5.0)	0 (0.0)	1 (5.0)	
**Aquatic activity featured**							0.049
Surfing	5 (5.1)	1 (5.3)	4 (20.0)	0 (0.0)	0 (0.0)	0 (0.0)	
Relaxing around waterways	2 (2.0)	1 (5.3)	0 (0.0)	0 (0.0)	0 (0.0)	1 (5.0)	
**Use of emojis**	51 (51.5)	16 (84.2)	1 (5.0)	16 (80.0)	13 (65.0)	5 (25.0)	<0.001
**Tags**							<0.001
Individual	7 (7.1)	3 (15.8)	0 (0.0)	0 (0.0)	2 (10.0)	2 (10.0)	
Company or brand	18 (18.2)	5 (26.3)	0 (0.0)	10 (50.0)	0 (0.0)	3 (15.0)	
**Advertising themes**							
Association with lifestyle	24 (24.2)	4 (21.1)	6 (30.0)	6 (30.0)	2 (10.0)	6 (30.0)	0.494
Association with hobbies and interests	33 (33.3)	2 (10.5)	3 (15.0)	9 (45.0)	6 (30.0)	13 (65.0)	0.001
**Marketing elements**							
Celebrities or sportspeople	8 (8.1)	1 (5.3)	1 (5.0)	1 (5.0)	0 (0.0)	5 (25.0)	0.037
Sponsorship or collaboration	23 (23.2)	1 (5.3)	0 (0.0)	9 (45.0)	0 (0.0)	13 (65.0)	<0.001

^a^ Differences between brands assessed using Chi-Square test.

## Data Availability

Codebook available on request.
